# Occupational Safety and Health Training for Undergraduates Nursing Students: A Spanish Pilot

**DOI:** 10.3390/ijerph17228381

**Published:** 2020-11-12

**Authors:** Esther Vaquero-Álvarez, Antonio Cubero-Atienza, María Pilar Martínez-Jiménez, Manuel Vaquero-Abellán, María Dolores Redel-Macías, Pilar Aparicio-Martínez

**Affiliations:** 1 SRH Kliniken Landkreis Sigmaringen, Hohenzollernstraße 40, 72488 Sigmaringen, Germany; esther.vaquero@srh.de; 2 Departamento Ingeniería Rural, Ed Leonardo da Vinci, Campus de Rabanales, Universidad de Córdoba, 14071 Córdoba, Spain; ajcubero@uco.es (A.C.-A.); ig1remam@uco.es (M.D.R.-M.); 3 Applied Physics, Radiology and Physics Medicine Department, Albert Einstein Building, Campus de Rabanales, Universidad de Cordoba, 14014 Cordoba, Spain; fa1majip@uco.es; 4 Simulation Models in Energy, Transport, Physics, Engineering, Occupational Hazard Researcher Group, Junta de Andalucía, and Dpt. Applied Physics, Albert Einstein Building, Campus de Rabanales, Universidad de Cordoba, 14014 Cordoba, Spain; 5 GC12 Clinical and Epidemiological Research in Primary Care, Instituto Maimónides, Campus de Menéndez Pidal, Universidad de Córdoba, 14071 Córdoba, Spain; en1vaabm@uco.es; 6 Departamento de Enfermería, Fisioterapia y Farmacología, Campus de Menéndez Pidal, Universidad de Córdoba, 14014 Córdoba, Spain

**Keywords:** prevention culture, continuous training, web platform, nursing students

## Abstract

Most of blood borne and airborne pathogens are highly contagious, harmful and have prevalence among healthcare workers. In this group, healthcare students, especially nursing undergraduates, have even higher risk to be exposed and suffered a contagious accident. One of the main pillars to prevent exposure to such pathogens and decrease accidents seems to be through education. A prospective observational educational research focused on quantifying the students’ knowledge, and prevention culture was carried out. The educational approach based on the development of a technological tool, its integration in the students’ education, and posterior assessment. The Chi-square, ANOVA, Kruskal–Wallis, Man–Whitney U, and Spearman correlations were used to determine the effect of such educational methodology. The results, previous to the integration of the educational approach, showed differences between the elementary and proficient knowledge and correct procedure in each academic year (*p* < 0.05), being the best year the third academic year. The mean of elementary knowledge among second year students after the inclusion of the educational methodology improved for 2017/2018 with a mean of 7.5 (1.11) and in 2018/2019 with 7.87 (1.34). This study argued that the educational approach proposed could improve the prevention culture and knowledge among students and future healthcare professionals.

## 1. Introduction

Since the first time that biological exposure to blood was studied by Ramazzini [1], more pathogens have been described as hazardous, being up to 44% of them viral [2]. Most of the current harmful microorganisms are blood borne, such as human immunodeficiency virus (H.I.V.), and airborne pathogens, such as tuberculosis, being highly presented in the daily day of healthcare workers [3,4]. These workers are at risk of being exposed to diverse pathogens, from mortal (i.e., Ebola) to highly contagious viruses (i.e., Sars-covid-19) via material or surfaces and corporal fluids [5,6].

This threat among healthcare workers continues to be presented and has economical and health repercussions in the healthcare systems and the workers’ physical and mental health [5,6]. In order to decrease this hazard, serval educational programs have been created and implemented to raise awareness among healthcare workers regarding risk and prevention, including hand washing or disposal in specific resistant containers [7]. At the same time, organizations and political structures, such as National Health System in England [5], have created prevention policies and updated guidelines to decrease the jeopardy of exposure among healthcare workers, protect their health, and concise these workers [8,9,10].

Among the measures taken by the organizations, the education (initial or continuous training) of their healthcare worker has been of the main pillars to prevent biological exposure and accidents [11]. Despite the modifications and inclusion of initial and continuous training carried out during more than two decades, the level of knowledge and compliance with universal precautions procedures continues to be limited [12,13]. This difficulty in achieving optimum levels of knowledge seems to be linked to the reduced safety culture [14]. The safety culture, which can be defined as values shared among workers regarding what is considered necessary among the healthcare workers, has been defined as a key point for occupational and health measures in different working areas [14]. One stage of the workers’ life that the learning process is more dynamic and adapting to changes is in the university years [8].

As future young workers, healthcare students are more drive to engage themselves in risky situations that they are not prepared to face, based on their willingness to take on challenges and more responsibilities. Moreover, the students rarely received information regarding the rate of the biological accidents that they had suffered during the practice [15]. Among the undergraduate healthcare students, the nursing students have a higher risk of exposure to pathogens because of their direct contact with the patients, lack of knowledge and safety culture, and more willingness to take risks [16]. Furthermore, data about biological accidents among nurses’ students continues to reduce, except from previous studies carried out in other countries, such as Italy [17,18] or China [19]. Moreover, only a few researchers have recently studied the incidence of biological accidents, knowledge and safety culture among nursing students in Spain [20].

## 2. Background

The evaluation of knowledge among healthcare students has been considered an upholder at improving their education and their safety culture, resulting in compromising the occupational health and security measures taken by the students [21]. The development of new educational methodologies [22] to improve the training and knowledge about preventing an accident and protocols post-exposition are a main key to improve the students safety culture. Simultaneously, educational institutions and researchers have created models and new methodological approaches based on computers and other information and communication technologies (I.C.T.s) to be integrated in the classroom [12]. These new methodologies search to adapt and develop platforms or applications via gamification or interactive environments, such as virtual reality, according to the organizations, students or professors’ needs [23,24]. All these tools are integrated into a platform or used individually, allowing ubiquitous, electronic or blended learning [25]. Previous works have accomplished several functional software applications available for mobile devices focused on nursing students [24,26].

Therefore, new teaching methodologies, based on ICTs and focused on improving the training and information about biological accidents and prevention, holds a great importance for nursing students [27]. These technologies were created to develop tutorials, games, virtual laboratories, videos, simulations, and virtual reality [24,27]. These technological and educational tools developed in computer-based training have four pillars to achieve their intended goal (feedback, appealing experience, creative design, and assessment of designed program) [28]. These pillars seem to positively improve the knowledge and training educational approaches via applications [28].

Nevertheless, current platforms, focused on risk prevention and occupational training and for undergraduate nursing students, continues to be lacking, which could make more difficult to improve their safety culture. Despite the incidence, low rate of prevention measures compliance, and safety culture [29], the current nursing students seemed not to be a priority, being included in the articles more as secondary actors [17]. Nevertheless, the education, aptitude, and knowledge of the undergraduate nursing student will define them as healthcare workers, and the future of the safety culture in this specific group [30].

Based on the previous statements, the current study had as main objective to determine the efficacy of the platform created, as a training tool, to improve the knowledge and safety culture among nursing students. In addition, the other secondary objectives were to analyze the state previous to the inclusion of the training tool, which was framed in an educational methodology, and the students’ opinion.

## 3. Materials and Methods

The current study presents a prospective observational educational research focused on quantifying the students’ knowledge and prevention culture after introducing the educational approach based on a blended learning. In addition, cross-sectional studies were carried out to determine the students’ knowledge and occupational culture’s consecutive measures and posterior knowledge and opinion after including the platform.

### 3.1. Educational Approach

#### 3.1.1. Creation of the Platform

**Figure 1:**
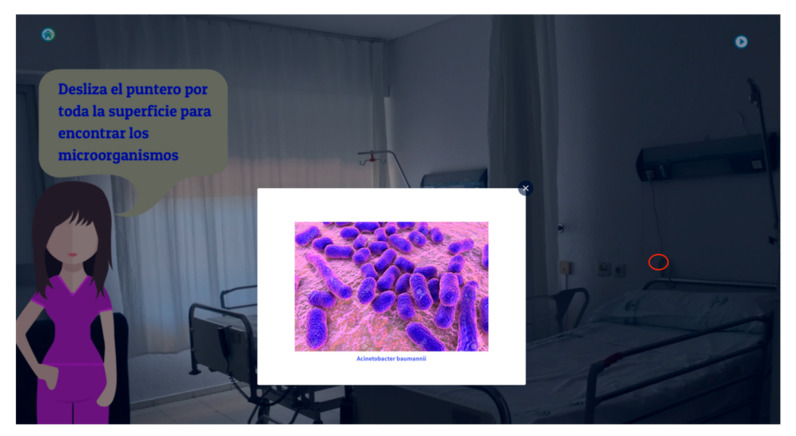
Example of a game in which the students need to locate the pathogens’ situation around the working environment. Note: the circle colored in red is the light cable where the pathogen is placed.

The platform (https://www.uco.es/investiga/grupos/LVRiesgosLaborales/formacion-sanitaria/) has been created using CakePHP as the primary programming language, and for the architecture pattern, it uses the control view model. Other languages, such as HTML5, CSS3, JavaScript with jQuery, JSON, and Ajax, were used. The design diagram mainly divided the coding into three layers: the model, view, and controller. This structure allows useful, resourceful, adaptable, and friendly software following the principle of “at any place and at any time”. This platform was divided into different sections depending on the user, administrator, and system programmer. This web was structured into tutorials, e-games, image galleries, valuation surveys, virtual laboratories, and help. The tutorials include a title, short description, and an image. The e-games was created mainly by using HtmL5, resulting in a more playful, visual, and short data training. An example of the games (Figure 1) focused on finding airborne and blood borne pathogens, visually appealing and more effective. The image galleries show a title, short description, and links to other webpages or further information. The valuation surveys were based on the Kahoot program. This survey was used during the training and information sessions in the theoretical subjects and clinical practice before the actual practice. Finally, the virtual laboratory focused on creating an interactive environment being accessible at any time. Moreover, a connection to social networks, such as Facebook and Twitter, were included. These sections were developed to integrate the primary information, images, and straightforward explanations that the student would need.

**Figure 2:**
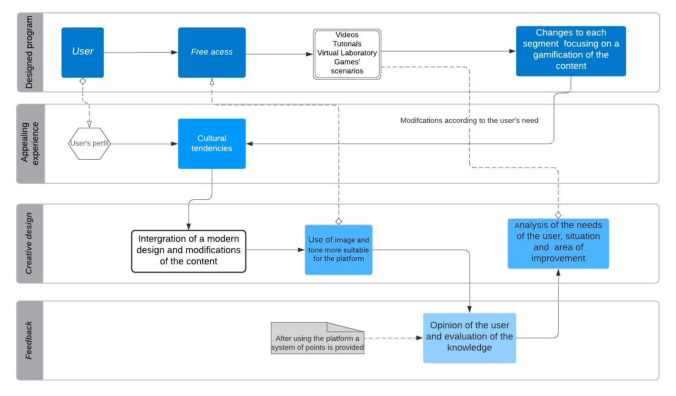
Flowchart of the integration of the fourth pillars in the structure of the educational methodology**.**

The platform creation was based on the fourth pillars (Figure 2) relating to gamification as an effective method for adapting and evaluating this platform as a prevention learning tool. This e-learning system’s design focused on stimulating knowledge acquisition and sought to apply a more dynamic teaching process being modified according to create a more appealing experience for the user.

**Figure 3:**
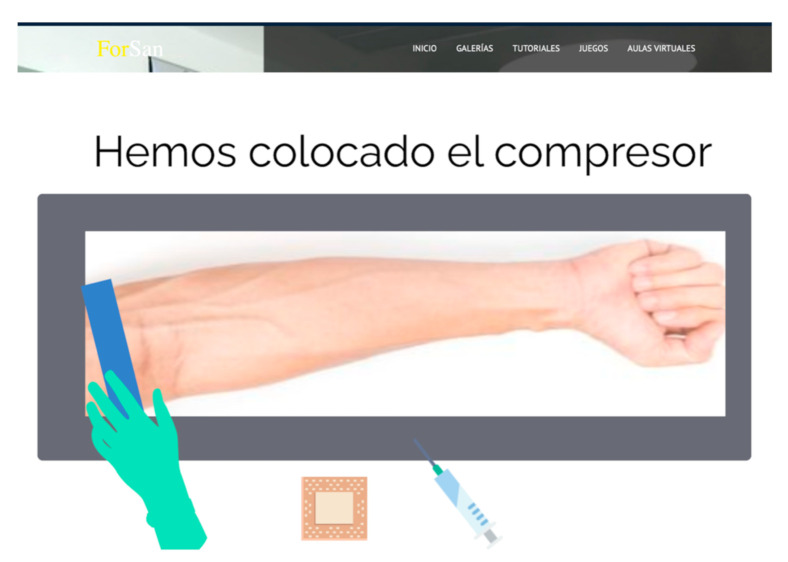
Virtual laboratory with the last modification following the impossibility to access patients**.**

Additionally, the platform’s design allowed several modifications to its content and the user’s needs. An example of this is the virtual laboratory last modified in May 2020 to provide a more complex intravenous puncture situation since the students could not access patients (Figure 3).

#### 3.1.2. Educational Paradigm and Procedure

The educational paradigm was based on combining in the classroom of face-to-face learning experiences with the platform created as an online learning experience, which can be defined as blended learning [31]. This educational paradigm focused on a quantitative or empiric approach, including the immediate results and the students’ opinion, eliminating the qualitative approach [32], following the theoretical models of blended learning [31]. This approach was selected to achieve the theoretical framework of the blended learning paradigm, focusing on exploring the state of the safety culture previous the use of the platform and possible associations [31]. The evaluation method was the context, input, process, and product (C.I.P.P.) method [33], by which five specialists in the prevention of occupational risk and engineering determine the area of improvement. These improvements focused on coding and structure being used in the platform.

For the students’ acquirement of knowledge and skills, the teachers were presented in the classroom when it was presential to explain and use the platform, creating an assisted blended learning. However, the platform’s versatility allows modification of the learning process from blended to virtual learning, as it happened with the pandemic and posterior lockdown [34]. Additionally, the students could access freely and have feedback with the teacher via the Moodle platform. This is why, for this educational process to be successful, the teachers provided help and support in acquiring prevention knowledge through communication and obtaining feedback on the students’ progress and their way of working. Finally, the students carried out a test to evaluate their knowledge after using the educational platform. The teaching and training were carried out during the first and second semesters. After the clinical practices, the students responded to an interactive survey in which the students’ knowledge about prevention was measured. The only exception was in the second semester of the course 2019/2020, in which the clinical practices were paused.

### 3.2. Determination of the Students’ Knowledge

#### 3.2.1. Data Collection

Before completing the platform, a sample of nursing students from different academic years was recruited, who voluntarily accessed the survey online during May and June in 2016. This recruitment sought to determine each academic year’s level of knowledge and possible lack of safety culture. The survey was distributed after ethical approval from the Ethics Committee of the University in 2015 (Reference 428). The survey, distributed at the end of the clinical practice in the second semester, included the description, objective, and the consent to participate that student signed. The nursing students received several theoretical practices and simulations before the clinical practices started. These students’ information and training focused on prevention measures, protocols, seroconversion incidence, and post-exposure procedures. It is important to note that the European credit for a nursing degree corresponds to 20 h/credit. Each academic year was formed by 120 students (110–130), although the students from the first year were excluded from the study. The nursing students were from the second, third, and fourth academic year, that belonged to the Medicine and Nursing School and had already been in hospital practices.

The platform’s assessment, focusing on design and appeal, was carried out from March to April 2017. These students chosen for the evaluation of the structure were from the second year, that did not receive the prevention training with the platform, and they evaluated the initial platform by pointing out improvements and possible changes that could be implemented. The posterior analysis received the approval of the updated protocol from the reference Ethics Committee in 2019 (Number 288, Reference 4258). The educational approach’s estimation based on a survey of students’ knowledge, being carried in the following years up to 2020 (May 2020), evaluated a total of 267 students for the three years. Finally, the students notified whether they did or did not suffer a biological accident, except for the course 2019/2020, the students did not have an accident because of the lack of clinical practices. The difference between having a higher level of knowledge and not experiencing a biological accident was studied. The results were compared to the knowledge and incidence of biological accidents from students between courses (2016/2017, 2017/2018, 2019/2018, and 2019/2020).

#### 3.2.2. Instruments

Before the inclusion of the educational technology, a survey that combined the questionnaires created by Merino-de la Hoz et al. in 2010 and Orozco in 2013 was used to measures the students’ knowledge [35,36]. The survey was composed of 48 questions, based on 38 closed answer items and ten open answers. The ten available answer questions were focused on the type of biological accident, year of the accident, experience, and post-exposure measures taken. The response was one per student, without the option to retake the survey. The sample’s descriptive data were age, gender, academic year, undergraduate background, and working experience. The survey was segmented into three main blocks. The first block focused on knowledge regarding prevention actions and biological agents. In addition, this block is divided into two sections: elementary and proficient knowledge. The elementary knowledge was based on universal prevention measures such as hand washing or personal protection equipment. Differently, proficient knowledge focused on specific data regarding prevention, occupational safety, and risks, such as seroconversion or treatments. The second block focused on the correct procedures during the practices, such as following the isolation protocols or changing gloves. Finally, the final block centred on the incidence of biological accidents, the factors contributing to the accident, and the measures taken after the incident (Table 1).

The platform’s assessment was accomplished by filling an in-person survey and included their personal opinion about the platform, being carried out from March to April 2017. This survey was based on a five-point scale, from one (minimum) to five (maximum) and according to the work of Garret Jackson in 2006 and Lahti M et al., 2014 [37,38]. The survey was segmentized into three segments, the education received previously (effectiveness of this education received previously (E1), the efficacy of the training to prevent biological accidents (E2) and frequency of using such training (E3)), valuation of the platform (easy use of the platform (V1), the utility of the gallery (V2), the usefulness of the tutorials (V3), usability and practicality of the virtual laboratories and games (V4) and use of social networks in which were included news and updated information (V5)) and finally their opinion of the platform as an interactive training (games and practice methodologies (O1), tutorials and practice information (O2), virtual simulations (O3), graphics and visual tools (O4) and interactive learning methodologies such as the platform developed (O5)). Two additional questions were included. The first focused on the students’ satisfaction concerning education, and the second was based on the students’ opinions about including games or other methodologies for occupational safety education.

The survey to determine the knowledge of the students’ posterior to the use of the platform was based on the survey by Merino-de la Hoz et al. and Orozco [35,36], focusing on the ten questions regarding the elementary knowledge. These surveys were adapted using “Kahoot!” an interactive software to fill in the survey or Moodle platform, obtaining a sample of approximately 110 students per academic year, except for 2020, that only 48 students filled it.

The programs used were Excel version 2017 (Microsoft Corporation, Redmond, WA, USA) and S.P.S.S. program version 25 (IBM SPSS Statistics, Armonk, NY, USA) for the statistical analysis. All the data was saved in a cloud available only to the researches. For the sample calculation, the E.P.I.D.A.T. version. 4.2. (Servicio de Epidemioloxía de la Dirección Xeral de Saúde Pública del Servicio Galego de Saúde (S.E.R.G.A.S.), Galicia, Spain) was used. The number of students was determined from an expected proportion of 30%, a 9% precision, and a confidence level of 95%. The initial predicted size was 100 students, though the actual size obtained was lower. The sample size for the validation of the students’ opinions was calculated from a standard deviation of 1.5, a 5% precision, and a confidence level of 95%.

#### 3.2.3. Statistical Analysis

The data were analyzed, and the normalization was studied using the Shapiro–Wilk test for the data before the inclusion of the educational approach. This analysis showed that the variable elementary knowledge was normalized, though the remaining variables were not standardized. The correct procedures variable was transformed into a quantitative variable based on a scale of 10, and a global variable focused on global preventive knowledge was created using the score obtained from the elementary and proficient preventive knowledge. The descriptive and frequencies were studied individually for each year, and the chi-test was used for assessment of the qualitative variables, i.e., the incidence of biological accidents and academic year. The student t-test was applied to knowledge, ANOVA test of variance for the academic years, and Kruskal–Wallis, Man–Whitney U, and Spearman correlations were used for comparisons.

The analysis of the comparison of each course (2016/2017, 2017/2018, 2019/2018, and 2019/2020) focused on frequencies and chi-square to determine the difference between each course, elementary knowledge of the students, and incidence of biological accidents. Additionally, for the courses, after the inclusion of the platform, being 2017/2018, 2019/2018, and 2019/2020, each correct answer was measured via scores, resulting in a mean of correct answers and standard deviation.

## 4. Results

The initial analysis of the data from the 2016/2017 course showed that only 22.6% of the population carry out the survey, representing 80 students out of the 354 sent the survey. This analysis also showed that 81.3% were women aged between 22 and 23, with an undergraduate background in health (45%), i.e., laboratory technicians, and 11.3% of the sample was working. The students were 27.5% from the second year, 43.9% from the third year, and 28.7% from the fourth year—87.5% from Spain, 10% from Portugal, and 2.5% from Eastern countries, i.e., Poland. Furthermore, 13 students suffered a biological accident, although the remaining data about such an accident was obtained for 12 students.

The correct response frequency between the elementary and proficient knowledge and correct procedure in each academic year was different (Table 2). The data showed a low level of correct answers according to knowledge and proper occupational safety and health measures among students, independently to the academic year. The 3rd presented had a higher answered prevention knowledge and occupational safety and health (O.S.H.) measure than the second and fourth academic years. The students from the third had tighter confidence intervals compared to the second and fourth year, with the lower interval set on four. The students’ global knowledge was sufficiently obtaining the maximum in the third, with tighter intervals compared to the second and fourth academic year (Table 2). In addition, the higher value of O.S.H. measures was obtained in the third year with a 0.8 difference between the intervals. Furthermore, the maximum values for elementary and proficient knowledge were obtained in the third year (maximum = 10; maximum = 6), followed by the second year for the elementary knowledge (maximum in elementary knowledge = 9; maximum in proficient knowledge = 4) and the fourth-year or proficient knowledge (maximum in elementary knowledge = 8; maximum in proficient knowledge = 5).

The relationship between the elementary and proficient knowledge and correctly carrying out the O.S.H. procedures was analyzed regarding the students’ academic year (Table 3). All variables’ data were studied between groups, showing a significant difference between elementary and proficient knowledge (*p* < 0.05). The results showed a difference between groups (second, third and fourth academic year) and the elementary and proficient knowledge, although, for the correct procedure, no significant difference was found among the groups (*p* > 0.05). The outcomes of this examination showed higher levels of correct answers regarding proficient knowledge in the third year compared with the second academic year (*p* < 0.05). This result was similar when compared second and fourth for the proficient and elementary knowledge, showing higher correct answers in the students from the fourth to the second academic year (*p* < 0.01). The comparative between the third and fourth showed no significant differences for the proficient knowledge (*p* > 0.05) and, in contrast, higher differences for the elementary knowledge (*p* < 0.01). Although the correct O.S.H. measures were not linked to a higher probability of suffering a biological accident, the O.S.H. was associated with higher notions of elementary knowledge (*p* < 0.05).

The frequencies presented 9.1% of second-year students, 14.3% third year, and 26.1% fourth had a hazardous accident with exposure to an airborne or bloodborne pathogen. Nevertheless, most of the students experienced this accident in their second (38.5%) or fourth academic year (38.5%), being less common to have it in third year (15.4%). The correlations showed a significance relationship between having a biological and, accident elementary (Spearman’s = −0.29; *p* < 0.01) and proficient (Spearman’s = 0.27; *p* < 0.05), and correct procedures (Spearman’s = −0.23; *p* < 0.05). Nevertheless, the mean of correct response comparing safety culture (knowledge and correct procedure) was insignificant for suffering (mean = 5.2) or not a biological accident (5.6) (*p* > 0.05). Another analysis focused on being a graduated health technician, and a lower probability of a biological accident was carried out, showing significant differences between being a graduated and not suffering biological accident (chi-square = 7.13 and *p* < 0.05).

Out of the students that indicated an incident (16.3%), only 92% included the type of accident, zone, cause, notification, and medical follow-up. The type of biological accident (N = 12) and the reason for the accident were studied. The most frequent accident was needlesticks (41.7%), followed by sharp injuries (33.3%), blood exposure (16.7%), and cuts (8.3%). The zone more common was fingers (83.3%), followed by arms (8.3%) and face (8.3%), and the most frequent cause was lack of knowledge or practice (41.7%), followed by rush (33.3%), inadequate use or knowledge regarding prevention instruments (16.7%) and carelessness (8.3%). The results showed that the most common cause in the second year was a lack of knowledge regarding the procedures (2/4). Meanwhile, the cause more common in the third year was the lack of knowledge regarding the specific procedures (2/3). Finally, in the fourth year, the reason for the accidents was the accident occurred.

**Figure 4:**
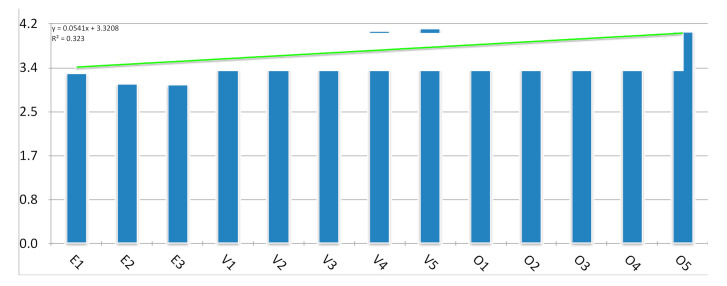
Assessment of the platform created based on the students’ opinion**s.**

Besides, the analysis of the platform carried out in 2017 (N = 40 students from the second academic year) showed a positive opinion regarding the prevention education received (3.1/5), the platform created as an interactive prevention intervention (3.9/5), and the students’ opinion about this technological tool for training (3.8/5). These students ranged from 19 to 20 years old (medium = 20 years old), analyzing the platform previous their first clinical practice in the hospital. Out of the 40 students, 36 were women with previous healthcare professional training (11.1%). The most common described was “adequate and helpful” (68%). Each variable of the students’ assessments regarding the platform was studied, showing the lowest value the previous training regarding O.S.H. measures and prevention (E3) (Figure 4). Figure 4 showed how social media (V5) had a higher positive evaluation (4.15). The next higher evaluation was the virtual laboratories and games (V4) (4.05), described as highly usable and appealing. Out of the 120 students asked about the platform, and the O.S.H. training, only 17% indication an acceptable grade of satisfaction with the O.S.H. education received. The students’ responses about the interactivity and possible use of the platform learning methodologies obtained the maximum ratings (5/5). Almost all of the students (97.2%) indicated their preference for including this technology, mainly through games, by which the platform’s content was modified.

Finally, the comparative analysis between the results of the survey for students on the second academic year in the course 2017/2018 (N = 111) and 2018/2019 (N = 110) showed significant differences in elementary knowledge (*p* < 0.01) and biological accidents (*p* < 0.05) when compared to the course 2016/2017. The means of correct answers regarding the elementary knowledge suffered an increase of a minimum of 0.2 points, although the standard deviation increased. In this sense, in 2017/2018, the mean was 7.5 (1.11) and in 2018/2019 was the best with 7.87 (1.34). The frequency of correct answers for the survey was higher in 2018/2019 (70.3%) than in 2017/2018 (65.2%). The elementary preventive knowledge was also analyzed for 2019/2020 (N = 60), showing a mean of correct answers of 7.32 (1.04), being almost equal to the course 2016/2019(*p* > 0.05). In biological accidents, the frequency of students who suffered a biological accident in the second academic year decreased by up to 1.9%.

## 5. Discussion

The results seemed to reflect on how the educational paradigm improved the student’s learning and possibly improved the prevention culture’s base. The most prominent finding to emerge from the analysis is that these technologies seemed to be useful as a complementary prevention tool for improving knowledge and decreasing biological accidents. This result matches the results from Bejan et al. [39] that indicated how the I.C.T.s improve the acquisition of safety-related knowledge among students even after a year of receiving training with I.C.T.s. Despite the initial results, an outstanding outcoming was that using only a virtual methodology without the student in the classroom seemed to be less effective than using the classroom technology with the students. These results matched the results from Kintu et al. in 2017, that concluded how the combination of technical quality, online tools, and face-to-face support increase the results and even satisfaction [40].

The results of safety culture, knowledge, and correct procedures showed medium and even low levels. The analyses showed that higher levels of knowledge and frequency of correctly performing the procedures during the clinical practices were linked to lower biological accidents. These results seem to match the findings of Wang et al. that proved how, with previous training, students had more prevention knowledge and had better occupational safety [41]. Moreover, these results are similar to be studies that highlighted the insufficient knowledge that healthcare workers have regarding O.S.H. measures and safety culture [15].

The O.S.H. measures did not improve with the academic years’ pass, rather the opposite, which could be explained as an integration of the ideas from the healthcare workers’ safety culture, whose actions or procedures are based on technical repetition and less guided by protocols or prevention measures [42,43]. During the clinical practices, riskier behaviors were linked to the exposure to airborne and blood pathogen, which was concerned with previous studies that linked risky behaviors and a higher probability of needlesticks and sharp injuries [18,19].

Another important finding was that elementary and proficient knowledge was related to suffering exposure to a pathogen. The result presented a positive correlation between proficient knowledge and biological accidents, indicating how students with higher knowledge regarding prevention or occupational safety suffered from more probability of biological accidents. These results are similar to those based on nurses working in hospitals that indicated a technical and more in-depth understanding of the prevention measures but suffered accidents [44]. These results may be related more to safety culture since the knowledge is presented, but the actions or significance given to the knowledge continues to be dismissed [14]. Other impressive results were the frequency of biological accidents that was lower than other studies, like Zhang et al., that indicated up to 60.3% of nursing students suffered a needle stick or sharps injuries [19]. A European study recently indicated up to 14.8% of biological accidents among healthcare students and residents [17]. This variation of frequency of biological accidents might be related to the students’ range or the previous training, which could explain the similarities between the Italian and the current study. Based on these results, the safety culture and occupational safety and health measures could be limited among students, although protocols or new methodologies have been created [45].

The platform created was evaluated as a competent preventive tool, although the most exciting finding was that the platform’s features with better valuation were the sections based on feedback and gamification. These results seem to match previous publications that stated the positive outcomes and valuation of such technologies, especially games as simulations to real scenarios and improving the O.S.H. measures among workers [46].

An explicit limitation of this study is that the sample of students who incorporated the surveys showed little interest from the students regarding improving preventive measures. This manuscript presents a pilot study that may result in a more in-depth analysis of biological accidents and related factors. In addition, another limitation was that the platform is only available in Spanish. Moreover, another source of uncertainty regarding the findings is the possibility of being transferred prevention knowledge to second-year students. These findings may be somewhat limited by the method of analysis and recruitment of the data. Finally, another limitation of the study was the time skipped between obtaining the biological data and current importance.

Further research would explore the inclusion of simulations via role play and virtual reality, described as highly significant (end-life, serious games), as interactive training combined with the technology created and technical information.

## 6. Conclusions

This paper has argued that new teaching methodologies based on I.C.T.s as a blended model could improve the prevention culture and knowledge among students and future healthcare professionals.

In addition, based on the study previous to the inclusion of the educational paradigm, it has been discussed how the exposure to airborne and bloodborne pathogens might be related to students’ knowledge and carry out correctly the O.S.H. measures. This study has identified that the ideal year to adequate and highlight the O.S.H. measures would in the beginning of the half of the degree, achieving the best results in the third academic year. This result was a key point, since the educational methodology was decided to take place in the second year to provide adequate knowledge since the early stages of the learning process of the nursing students. In general, the results have discussed how the elementary preventive knowledge and correct O.S.H. measures during the clinical practice procedures might be protective factors against exposures to pathogens. These data supposed a strong base for improving the students’ knowledge and O.S.H. measures via the platform and using preferable a blended method.

After in the inclusion of the platform, its assessment showed that students’ opinions regarding the inclusion of this educational approach based on technology was satisfactory. The posterior analyses of the elementary knowledge and incidence of biological accidents demonstrated the platform’s potential as a useful and practical preventive tool for the nursing students, but healthcare workers could also use it for their continues training. The findings of this platform’s probable effectiveness in the decrease of the exposures may contribute in several ways to the prevention of occupational accidents. Additionally, the findings provide further information regarding new interactive learning methodologies and provide a basis for future investigation, based on further gamification, virtual reality, and artificial intelligence.

## Figures and Tables

**Table 1:** Structure of the survey used on the students.

Blocks	Description	Questions	Examples
Prevention of Knowledge	Elementary Knowledge	Ten questions about elementary prevention measures	Select the prevent universal actions
	Proficient Knowledge	Ten questions about specific information	Human inmunodeficiency virus (HIV) seroconversion rate
Correct Procedures	Procedures during the practices	Eight questions about preventing universal measures and avoiding incorrect procedures	How often do you re-encapsulated needles?
Biological accident	Having or not a biological accident and procedure post-exposure	Ten questions about the biological accident and procedure post-exposure	What type of biological accident did you have?

**Table 2:** Descriptive analysis of each year of the 80 students.

Academic Year	Number of Students	Elementary Knowledge		Proficient Knowledge		Occupational Safety and Health Measures		Global Prevention Knowledge	
		Mean (S.D.)	CI 95%	Mean (S.D.)	CI 95%	Mean (S.D.)	CI 95%	Mean (S.D.)	CI 95%
Second Year	22	7.3 (1.0)	6.8–7.8	2.9 (0.8)	2.5–3.3	6.6 (1.3)	6.0–7.2	5.1 (0.7)	4.8–5.4
Third Year	35	7.4 (1.2)	7–7.8	3.6 (1)	3.2–3.9	7.3 (1.2)	6.9–7.7	5.5 (0.8)	5.3–5.5
Fourth Year	23	6.3 (0.9)	5.8–6.7	3.7 (1)	3.1–4.1	5.0 (0.8)	4.6–5.3	5.0 (0.8)	4.6–5.3

**Table 3:** Relationship between knowledge and correct procedure according to each academic year.

Blocks	3rd Academic Year	2nd and 3rd	2nd and 4th	3rd and 4th
Elementary Knowledge	<0.01	>0.05	<0.01	<0.001
Proficient Knowledge	<0.01	<0.01	<0.05	>0.05
Correct Procedure	>0.05	>0.05	>0.05	>0.05
